# Supplementary material for: Effects of guanylurea, the transformation product of the antidiabetic drug metformin, on the health of brown trout (Salmo trutta f. fario)
Source: PeerJ. 2019 Jul 11;7:e7289. doi: 10.7717/peerj.7289 (PMC6626654; doi:10.7717/peerj.7289)
Supplement: Supplemental Information 1 [file peerj-07-7289-s001.docx]

# Supplement

## Measurement of the concentrations of guanylurea in the exposure medium

Tab. S. 1: Operating parameters of the quadrupole time of flight mass spectrometer (QTOF-MS) for the quantification of the guanylurea concentrations in the test medium.

| **Parameter** | **Set point** |
| --- | --- |
| Gas temperature | 150 °C |
| Gas flow | 16 L/min |
| Nebulizer | 35 psi |
| Sheath gas heater | 400 °C |
| Sheath gas flow | 12 L/min |
| Capillary voltage | 3000 V |
| OctopolRFPeak | 750 V |
| Fragmentor voltage | 360 V |
| Scan Rate | 3 spectra/sec |
| Scan range | 60-1000 m/z |

Tab. S. 2: Mean guanylurea water concentrations of the test aquaria per treatment for the experiment with the larval brown trout exposed to guanylurea, measured with LC-MS

|  | 29.12.17  start | 22.01.18  before w.e. | 22.01.18  after w.e. | 22.02.18  before w.e. | 22.02.18  after w.e. | 15.03.18 before w.e. | 15.03.18 after w.e. | 17.04.18  end |
| --- | --- | --- | --- | --- | --- | --- | --- | --- |
| 0 µg/L | < LoQ | < LoQ | < LoQ | < LoQ | < LoQ | < LoQ | < LoQ | < LoQ |
| 10 µg/L | 10.0 | 7.7 | 8.0 | 9.0 | 9.6 | 8.3 | 8.6 | 10.4 |
| 100 µg/L | 115.0 | 126.9 | 112.0 | 114.8 | 111.1 | 120.6 | 114.4 | 123.3 |
| 1000 µg/L | 1238 | 1286 | 1211 | 1300 | 1290 | 1228 | 1257 | 1218 |
| LoQ= limit of quantification; before/after w.e.=before/after water exchange | | | | | | | | |

Tab. S. 3: Mean guanylurea water concentrations of the test aquaria per treatment for the experiment with juvenile brown trout exposed to guanylurea, measured with LC-MS

|  | 08.08.18  start | 21.08.18  before w.e. | 21.08.18  after w.e. | 05.09.18  before w.e. |
| --- | --- | --- | --- | --- |
| 0 µg/L | < LoQ | < LoQ | < LoQ | < LoQ |
| 10 µg/L | 11.81 | 11.55 | 11.54 | 12.08 |
| 100 µg/L | 135.3 | 134.1 | 129.1 | 131.4 |
| 1000 µg/L | 1416 | 1298 | 1308 | 1253 |
| LoQ= limit of quantification; before/after w.e.=before/after water exchange | | | | |

## Measurement of guanylurea in the tissue of brown trout larvae

We used solid phase extraction with a weak cation exchange functionalized polymeric material (Strata XCW) for the determination of internal concentrations of guanylurea. Therefore, we homogenized frozen fish tissue with mortar and pestle under liquid nitrogen. Afterwards, aliquots of 100 mg of the homogenized sample were weighed into a 2.5 mL tube (Eppendorf, Germany) and 1.5 mL water (Cromasolv, Merck, Darmstadt, Germany) were added. The sample was shaken for 30s using a Vortex mixer and the precipitate was centrifuged at 13.000g for 15 min. Afterwards the supernatant was transferred into an Eppendorf tube and was then ready for SPE extraction. Prior to the extraction the material was conditioned with 3x3 mL methanol followed by 3x3 mL of water (LC-MS grade). After equilibrating, 1 mL sample extract was loaded onto the cartridges. Elution of guanylurea was performed with 1 mL of methanol/acetonitrile mixture containing 2 % formic acid. The eluate was evaporated to dryness under a gentle stream of nitrogen and the concentrated residue was redissolved in 300 μL methanol. After filtration using a 45 μm PTFE filter (pore size 0.45 μm, Chromafil, Macherey-Nagel, Germany), the samples were analysed by CE–MS.

The CE separations were carried with an uncoated fused-silica capillary (length 80 cm, i.d. 50 µm). The samples were injected hydrodynamically by applying a pressure of 100 mbar for 10s. New capillaries were conditioned with 1 M NaOH for 15 min, water for 10 min and BGE for 15 min. Before each run the capillary was conditioned with BGE for 5 min. The CE capillary was kept at 25 °C during CE runs and a voltage of +30 kV was applied.

All analyses were performed using an Agilent CE 7100 interfaced to an Agilent 6550 iFunnel Q-TOF mass spectrometer (Agilent Technologies, Santa Clara, CA, USA) with an electrospray ionization source (ESI) assisted by sheath liquid interface. The composition of the sheath liquid was isopropanol/water (1:1, v/v) with 0.1 % FA. The sheath liquid was delivered by a 1260 isocratic pump (Agilent Technologies, Waldbronn, Germany) at a flow rate of 4 µL/min. The nebulizer pressure was set to 0.28 bar and the drying gas flow rate to 4 L/min. A fragmentor voltage of 175 V, a capillary voltage of −4.000 V, a skimmer voltage of 65 V and an octopole voltage of 750 V were used. The mass range was set to m/z 100–1.700, and the data acquisition rate was two spectra/s. For internal calibration purine, HP0321 and HP0921 (Agilent Technologies, Waldbronn, Germany) were used. Data analysis was accomplished using MassHunter software (Agilent Technologies, Waldbronn, Germany). Internal guanylurea concentrations were calculated based on a calibration curve between 5 and 50 μg/L and the recovery of the SPE method was determined to 84 %.

## Water quality parameters

Tab. S. 4: Water quality parameters of the experiment with brown trout larvae exposed to guanylurea

| **Guanylurea concentration** | **Replicate number** | **Oxygen concentrationt [mg/L]** | **pH [-]** | **Conductivity [µS/cm]** | **Temperature [°C]** | **Measurement time point** |
| --- | --- | --- | --- | --- | --- | --- |
| 0 µg/L | 1 | 10.92 | 8.26 | 490 | 7.6 | day 0 (start) |
| 0 µg/L | 2 | 10.95 | 8.28 | 490 | 7.5 | day 0 (start) |
| 0 µg/L | 3 | 10.95 | 8.28 | 490 | 7.5 | day 0 (start) |
| 10 µg/L | 1 | 10.88 | 8.33 | 487 | 7.7 | day 0 (start) |
| 10 µg/L | 2 | 10.95 | 8.35 | 490 | 7.3 | day 0 (start) |
| 10 µg/L | 3 | 11.02 | 8.32 | 491 | 7.1 | day 0 (start) |
| 100 µg/L | 1 | 10.96 | 8.32 | 488 | 7.4 | day 0 (start) |
| 100 µg/L | 2 | 11.03 | 8.33 | 487 | 7.0 | day 0 (start) |
| 100 µg/L | 3 | 11.05 | 8.37 | 487 | 7.1 | day 0 (start) |
| 1000 µg/L | 1 | 10.80 | 8.29 | 487 | 7.7 | day 0 (start) |
| 1000 µg/L | 2 | 10.93 | 8.30 | 486 | 7.1 | day 0 (start) |
| 1000 µg/L | 3 | 10.59 | 8.30 | 487 | 7.3 | day 55 |
| 0 µg/L | 1 | 10.87 | 8.21 | 466 | 7.5 | day 55 |
| 0 µg/L | 2 | 10.88 | 8.23 | 465 | 7.4 | day 55 |
| 0 µg/L | 3 | 10.87 | 8.28 | 466 | 7.4 | day 55 |
| 10 µg/L | 1 | 10.77 | 8.34 | 465 | 7.6 | day 55 |
| 10 µg/L | 2 | 10.82 | 8.40 | 465 | 7.3 | day 55 |
| 10 µg/L | 3 | 10.82 | 8.38 | 465 | 7.4 | day 55 |
| 100 µg/L | 1 | 10.83 | 8.41 | 462 | 7.5 | day 55 |
| 100 µg/L | 2 | 10.82 | 8.39 | 461 | 7.3 | day 55 |
| 100 µg/L | 3 | 10.85 | 8.39 | 462 | 7.2 | day 55 |
| 1000 µg/L | 1 | 10.75 | 8.41 | 462 | 7.6 | day 55 |
| 1000 µg/L | 2 | 10.79 | 8.43 | 461 | 7.3 | day 55 |
| 1000 µg/L | 3 | 10.83 | 8.45 | 462 | 7.3 | day 55 |
| 0 µg/L | 1 | 11.21 | 8.24 | 495 | 7.5 | day 110 (end) |
| 0 µg/L | 2 | 11.25 | 8.28 | 496 | 7.2 | day 110 (end) |
| 0 µg/L | 3 | 11.26 | 8.31 | 498 | 7.3 | day 110 (end) |
| 10 µg/L | 1 | 11.23 | 8.32 | 500 | 7.4 | day 110 (end) |
| 10 µg/L | 2 | 11.27 | 8.34 | 499 | 7.2 | day 110 (end) |
| 10 µg/L | 3 | 11.27 | 8.35 | 501 | 7.2 | day 110 (end) |
| 100 µg/L | 1 | 11.22 | 8.35 | 498 | 7.4 | day 110 (end) |
| 100 µg/L | 2 | 11.31 | 8.35 | 497 | 6.9 | day 110 (end) |
| 100 µg/L | 3 | 11.34 | 8.34 | 497 | 7.0 | day 110 (end) |
| 1000 µg/L | 1 | 11.28 | 8.34 | 499 | 7.3 | day 110 (end) |
| 1000 µg/L | 2 | 11.26 | 8.35 | 498 | 6.9 | day 110 (end) |
| 1000 µg/L | 3 | 11.17 | 8.33 | 499 | 7.2 | day 110 (end) |

Tab. S. 5: Water quality parameters of the experiment with juvenile brown trout exposed to guanylurea

| **Guanylurea concentration** | **Replicate number** | **Oxygen concentration [mg/L]** | **pH [-]** | **Conductivity [µS/cm]** | **Temperature [°C]** | **Measurement time point** |
| --- | --- | --- | --- | --- | --- | --- |
| 0 µg/L | 1 | 11.07 | 8.00 | 475 | 7.6 | day 0 (start) |
| 0 µg/L | 2 | 11.38 | 8.18 | 475 | 6.6 | day 0 (start) |
| 0 µg/L | 3 | 11.39 | 8.21 | 477 | 6.8 | day 0 (start) |
| 10 µg/L | 1 | 11.23 | 8.22 | 477 | 7.4 | day 0 (start) |
| 10 µg/L | 2 | 11.26 | 8.24 | 476 | 6.9 | day 0 (start) |
| 10 µg/L | 3 | 11.31 | 8.25 | 476 | 6.9 | day 0 (start) |
| 100 µg/L | 1 | 11.28 | 8.26 | 474 | 7.3 | day 0 (start) |
| 100 µg/L | 2 | 11.27 | 8.26 | 472 | 6.9 | day 0 (start) |
| 100 µg/L | 3 | 11.31 | 8.26 | 472 | 6.8 | day 0 (start) |
| 1000 µg/L | 1 | 11.33 | 8.27 | 474 | 7.1 | day 0 (start) |
| 1000 µg/L | 2 | 11.31 | 8.27 | 473 | 6.9 | day 0 (start) |
| 1000 µg/L | 3 | 11.33 | 8.27 | 474 | 7.0 | day 0 (start) |
| 0 µg/L | 1 | 11.31 | 8.37 | 516 | 7.4 | day 29 (end) |
| 0 µg/L | 2 | 11.57 | 8.4 | 519 | 6.7 | day 29 (end) |
| 0 µg/L | 3 | 11.59 | 8.42 | 521 | 6.7 | day 29 (end) |
| 10 µg/L | 1 | 11.53 | 8.44 | 521 | 7.0 | day 29 (end) |
| 10 µg/L | 2 | 11.57 | 8.45 | 526 | 6.7 | day 29 (end) |
| 10 µg/L | 3 | 11.61 | 8.44 | 525 | 6.6 | day 29 (end) |
| 100 µg/L | 1 | 11.51 | 8.45 | 522 | 7.2 | day 29 (end) |
| 100 µg/L | 2 | 11.59 | 8.46 | 516 | 6.5 | day 29 (end) |
| 100 µg/L | 3 | 11.62 | 8.45 | 518 | 6.4 | day 29 (end) |
| 1000 µg/L | 1 | 11.57 | 8.46 | 517 | 6.8 | day 29 (end) |
| 1000 µg/L | 2 | 11.55 | 8.46 | 518 | 6.5 | day 29 (end) |
| 1000 µg/L | 3 | 11.63 | 8.46 | 516 | 6.8 | day 29 (end) |

## Histopathological examinations

Qualitative examination of the samples with histopathological findings in the liver of brown trout (see Tables S. 6 and 7)

| **Dark grey** | **Grey** | **White** |
| --- | --- | --- |
| High glycogen content | Medium glycogen content | Low glycogen content |
| No macrophage agglomeration | Small macrophage agglomeration | Big macrophage agglomeration |
| Large cell size | Medium cell size | Small cell size |
| Bright cytoplasm | Medium cytoplasm | Dark cytoplasm |

Tab. S. 6: Detailed information for the histopathological findings in the liver of larval brown trout exposed to guanylurea (for the qualitative examination) (for information on grey scales see table above)

| **Larval brown trout** | **0 µg/L** | | | **10 µg/L** | | | **100 µg/L** | | | **1000 µg/L** | | |
| --- | --- | --- | --- | --- | --- | --- | --- | --- | --- | --- | --- | --- |
| Glycogen amount | 19 | 1 | 1 | 14 | 6 | 1 | 18 | 5 | 0 | 16 | 6 | 1 |
| Macrophage agglomeration | 21 | 0 | 0 | 20 | 1 | 0 | 23 | 0 | 0 | 22 | 1 | 0 |
| Cell size | 17 | 3 | 1 | 14 | 6 | 1 | 13 | 10 | 0 | 13 | 9 | 1 |
| Cytoplasm | 18 | 2 | 1 | 17 | 3 | 1 | 17 | 6 | 0 | 19 | 3 | 1 |

Tab. S. 7: Detailed information for the histopathological findings in the liver of juvenile brown trout exposed to guanylurea (for the qualitative examination) (for information on grey scales see table above)

| **Juvenile brown trout** | **0 µg/L** | | | **10 µg/L** | | | **100 µg/L** | | | **1000 µg/L** | | |
| --- | --- | --- | --- | --- | --- | --- | --- | --- | --- | --- | --- | --- |
| Glycogen amount | 5 | 6 | 8 | 4 | 11 | 4 | 9 | 9 | 2 | 5 | 10 | 6 |
| Macrophage agglomeration | 17 | 2 | 0 | 16 | 2 | 1 | 13 | 4 | 4 | 17 | 3 | 1 |
| Cell size | 2 | 12 | 5 | 3 | 12 | 4 | 5 | 12 | 4 | 2 | 14 | 5 |
| Cytoplasm | 2 | 7 | 10 | 4 | 11 | 4 | 5 | 11 | 5 | 4 | 9 | 8 |

Qualitative examination of the samples with histopathological findings in the kidney of brown trout (see Table S. 8)

| **Dark grey** | **Grey** | **White** |
| --- | --- | --- |
| Small vesicles | Enlarged vesicles | Hyaline droplets |
| No vacuoles | Small vacuoles | Large vacuoles |
| Intact hematopoietic tissue | Partly degenerated hematopoietic tissue | Degenerated hematopoietic tissue |
| Intact Bowman’s capsule | Partly enlarged Bowman’s capsule | Enlarged Bowman’s capsule |

Tab. S. 8: Detailed information for the histopathological findings in the kidney of juvenile brown trout exposed to guanylurea (for the qualitative examination) (for information on grey scales see table above)

| **Juvenile brown trout** | **0 µg/L** | | | **10 µg/L** | | | **100 µg/L** | | | **1000 µg/L** | | |
| --- | --- | --- | --- | --- | --- | --- | --- | --- | --- | --- | --- | --- |
| Vesicles | 11 | 3 | 4 | 11 | 2 | 6 | 12 | 1 | 6 | 14 | 3 | 3 |
| Vacuoles | 7 | 1 | 10 | 8 | 5 | 6 | 4 | 4 | 11 | 10 | 3 | 7 |
| Hematopoietic tissue | 18 | 0 | 0 | 19 | 0 | 0 | 18 | 1 | 0 | 20 | 0 | 0 |
| Bowman’s capsule | 16 | 1 | 1 | 18 | 0 | 1 | 18 | 1 | 0 | 20 | 0 | 0 |

## Statistical details

Tab. S. 9: Details for the statistical analyses of the experiments with larval brown trout exposed to guanylurea

| **Larval brown trout** |  |  |
| --- | --- | --- |
| Mortality | COX-regression | df=3; χ^2^=4.6405; p=0.2001 |
| Heart rate | Nested ANOVA | df_1_=1, df_2_=24; F=0.0133; p=0.9090 |
| Time to hatch | COX-regression | df=3; χ^2^=7.11E-9; p=1.0 |
| Body weight | Nested ANOVA | df_1_=3, df_2_=332; F=0.2065; p=0.8919; transformation via natural logarithm |
| Body length | Nested ANOVA | df_1_=3, df_2_=332; F=2.5073; p=0.0589 |
| Condition factor | Nested ANOVA | df_1_=3, df_2_=332; F=2.5092; p=0.0587 |
| Lipid peroxides | Welch-ANOVA | df numerator=3 /df denominator=63.139; p=0.2333; transformation via x^-0.5^ |
| Histopathology of liver | Likelihood-ratio-χ^2^-test | df=6; χ^2^=7.387; p=0.2865 |

Tab. S. 10: Details for the statistical analyses of the experiments with juvenile brown trout exposed to guanylurea

| **Juvenile brown trout** |  |  |
| --- | --- | --- |
| Mortality | COX-regression | df=3; χ^2^=2.7890; p=0.4253 |
| Body weight | Nested ANOVA | df_1_=3, df_2_=70; F=0.5674; p=0.6383; transformation via  x^-0.5^ |
| Body length | Nested ANOVA | df_1_=3, df_2_=70; F=0.3133; p=0.8157; transformation via  x^-0.5^ |
| Condition factor | Nested ANOVA | df_1_=3, df_2_=70; F=0.3431; p=0.7942 |
| Stress proteins | Nested ANOVA | df_1_=3, df_2_=70; F=1.1804; p=0.3235 |
| Histopathology of liver | Likelihood-ratio-χ^2^-test | df=9; χ^2^=8.117; p=0.5224 |
| Histopathology of kidney | Likelihood-ratio-χ^2^-test | df=9; χ^2^=6.715; p=0.6668 |
| Total distance moved | Nested ANOVA | df_1_=3, df_2_=24; F=1.8356; p=0.1676; transformation via natural logarithm |
| Mean velocity | Nested ANOVA | df_1_=3, df_2_=24; F=1.8356; p=0.1676; transformation via natural logarithm |

## CRED-criteria

| **CRED-criteria: Exposure of brown trout larvae to guanylurea** | | | | | |
| --- | --- | --- | --- | --- | --- |
| **1. General information** | | | | | |
| a. Purpose | | | Aim of the study was to investigate the effect of guanylurea on the health and development of brown trout larvae. | | |
| b. Endpoints | | | Mortality, body weight, body length, heart rate, hatching success, hatching rate, histopathological investigation of the liver, analyses of the lipid peroxide level and of the vitellogenin content (data not shown) | | |
| **2. Test design** | | | | | |
| a. Standard | | | Not performed according to a standard procedure | | |
| b. GLP | | | Not GLP-accredited | | |
| c. Controls | | | Laboratory negative control | | |
| d. Validity | | | The mortality of the control was 5.56 %. | | |
| **3. Test compound** | | | | | |
| a. Identification | | Guanylurea sulfate (CAS: 591-01-5) | | |  |
| b. Physico-chemical characteristics | | Water solubility= 50 g/L  (source: chemIDplus by toxnet) | | |  |
| c. Source | | TCI, Product Number: D0433; Batch Number: AKJLG | | |  |
| d. Purity | | >98% according to the Certificate of Analysis from TCI | | |  |
| e. Formulation | | No formulation, no impurities | | |  |
| **4. Test organism** | | | | |  |
| a. Scientific name | | *Salmo trutta* f. *fario* | | |  |
| b. Body weight/length | | Body weight: mean=0.502 g ± 0.13  Body length: mean=3.74cm ± 0.29 | | |  |
| c. Age/life stage | | From eyed egg stage until 8 weeks post yolk-sac consumption (larvae) | | |  |
| d. Reproductive condition | | Not in reproductive condition | | |  |
| e. Sex | | Not determinable | | |  |
| f. Strain/clone | | No defined clone | | |  |
| g. Source | | commercial trout farm (Forellenzucht Lohmühle, D-72275 Alpirsbach-Ehlenbogen) | | |  |
| h. Acclimatisation | | No acclimatisation | | |  |
| **5. Exposure conditions** | | | | |  |
| a. Schedule | | Semi-static design with water exchange twice a week | | |  |
| b. System | | closed | | |  |
| c. Test medium | | Filtered tap water (iron filter, active charcoal filter, particle filter) cooled to 7°C and aerated | | |  |
| d. Temperature | | Climate chamber set to 7 °C, measured at the beginning and the end of the experiment; mean=7.3 °C ± 0.2 °C | | |  |
| e. pH | | Measured at the beginning and the end of the experiment; mean=8.33 ± 0.05 | | |  |
| f. Hardness | | Not measured | | |  |
| g. Conductivity | | Measured at the beginning and the end of the experiment; mean=483 µS/cm ± 15 µS/cm | | |  |
| h. Dissolved oxygen | | Measured at the beginning and the end of the experiment; mean=11.00 mg/L ± 0.2 mg/L | | |  |
| i. Light intensity/quality | | 10 h : 14 h light:dark cycle; aquaria shaded from direct light with black plastic foil | | |  |
| j. Feeding | | Once per day with commerical trout feed 0.5 mm for the first 4 weeks, then 0.8 mm (INICIO Plus, Biomar, Denmark) | | |  |
| k. Aquaria | | 25 L glass aquaria filled with 10 L resp. 15 L of medium, covered with glass plane, silicone tubing, aerated with airstones (JBL ProSilent Aeras Micro S2) | | |  |
| l. Sand/sediment | | No sediment tested | | |  |
| m. Stock solutions | | Stock solution 1 (100 mg/L) prepared from 148.03 mg guanylurea sulfate in 1 dest. water, stock solution 2 (10 mg/L) produced from stock solution 1 via 1:10 dilution | | |  |
| n. Nominal concentrations | | 0, 10, 100, 1000 μg/L | | |  |
| o. Measured concentration | | Water samples were taken and analysed at the beginning and the end of the experiment as well as at three times during the experiments, before and after the water exchange (details in Paragraph 1) | | |  |
| p. Method | | HPLC-MS (QTOF-MS) (LoQ = 300 ng/L) | | |  |
| q. Duration | | 29.12.17 – 17.04.18; 110 days | | |  |
| r. Observations | | Mortality, behaviour, at the end of the experiment body length and body weight were determined and samples were taken for biochemical and histological analyses | | |  |
| s. Results | | Summary table in article | | |  |
| t. Biomass loading | | Mean= 1g/L | | |  |
| **6. Statistical Design and Biological Response** | | | |  |  |
| a. Replicates | Three replicate aquaria per test concentration | | |  |  |
| b. Number of organisms | 30 fish per replicate | | |  |  |
| c. Design | Three blocks, one replicate per treatment present in each block, arranged in randomized order | | |  |  |
| d. Statistical methods | Mortality and hatching rate: nested COX-regression, body length/mass, heart rate, lipid peroxide-level: nested ANOVA, histology: likelihood ratio test | | |  |  |
| e. Biological response | None | | |  |  |
| f. Dose-response | None | | |  |  |
| g. Statistical significances | None | | |  |  |
| h. Significance level | α = 0.05, in cases of multiple comparisons adjusted via sequential Bonferroni | | |  |  |
| i. Variability | Not estimated | | |  |  |
| j. Raw data | Provided on request | | |  |  |

| **CRED-criteria: Exposure of juvenile brown trout to guanylurea** | | | | | |
| --- | --- | --- | --- | --- | --- |
| **1. General information** | | | | | |
| a. Purpose | | | Aim of the study was to investigate the effect of guanylurea on the health of juvenile brown trout. | | |
| b. Endpoints | | | Mortality, body weight, body length, histopathological investigation of the liver and kidney, analyses of the Hsp70-Level and of the swimming behaviour | | |
| **2. Test design** | | | | | |
| a. Standard | | | Not performed according to a standard procedure | | |
| b. GLP | | | Not GLP-accredited | | |
| c. Controls | | | Laboratory negative control, hatchery control | | |
| d. Validity | | | The mortality of the control was 3.34%. | | |
| **3. Test compound** | | | | | |
| a. Identification | | Guanylurea sulfate (CAS: 591-01-5) | | |  |
| b. Physico-chemical characteristics | | Water solubility= 50 g/L  (source: chemIDplus by toxnet) | | |  |
| c. Source | | TCI, Product Number: D0433; Batch Number: AKJLG & WIA7F | | |  |
| d. Purity | | >98% according to the Certificate of Analysis from TCI | | |  |
| e. Formulation | | No formulation, no impurities | | |  |
| **4. Test organism** | | | | |  |
| a. Scientific name | | *Salmo trutta* f. *fario* | | |  |
| b. Body weight/length | | Body weight: mean=2.89 g ± 0.89  Body length: mean=6.49 cm ± 0.67 | | |  |
| c. Age/life stage | | Juveniles, approx. 9 months old | | |  |
| d. Reproductive condition | | Not in reproductive condition | | |  |
| e. Sex | | Not determinable | | |  |
| f. Strain/clone | | No defined clone | | |  |
| g. Source | | Commercial trout farm (Forellenzucht Lohmühle, D-72275 Alpirsbach-Ehlenbogen) | | |  |
| h. Acclimatisation | | Acclimatisation to lab conditions (water quality, temperature, pH) for two week | | |  |
| **5. Exposure conditions** | | | | |  |
| a. Schedule | | Semi-static design with water exchange twice a week | | |  |
| b. System | | Closed | | |  |
| c. Test medium | | Filtered tap water (iron filter, active charcoal filter, particle filter) cooled to 7°C and aerated | | |  |
| d. Temperature | | Climate chamber set to 7 °C, measured at the beginning and the end of the experiment; mean=6.9 °C ± 0.3 °C | | |  |
| e. pH | | Measured at the beginning and the end of the experiment; mean=8.33 ± 0.12 | | |  |
| f. Hardness | | Not measured | | |  |
| g. Conductivity | | Measured at the beginning and the end of the experiment; mean=497 µS/cm ± 23 µS/cm | | |  |
| h. Dissolved oxygen | | Measured at the beginning and the end of the experiment; mean=11.42 mg/L ±0.15 mg/L | | |  |
| i. Light intensity/quality | | 10 h : 14 h light:dark cycle; aquaria shaded from direct light with black plastic foil | | |  |
| j. Feeding | | Once per day with commerical trout feed (0.8 mm, Inicio Plus, Biomar, Denmark) | | |  |
| k. Aquaria | | 25 L glass aquaria filled with 15 L of medium, covered with glass plane, silicone tubing, aerated with airstones (JBL ProSilent Aeras Micro S2) | | |  |
| l. Sand/sediment | | no sediment tested | | |  |
| m. Stock solutions | | Stock solution 1 (100 mg/L) prepared from 148.03 mg guanylurea sulfate in 1 dest. water, stock solution 2 (10 mg/L) produced from stock solution 1 via 1:10 dilution | | |  |
| n. Nominal concentrations | | 0, 10, 100, 1000 μg/L | | |  |
| o. Measured concentration | | Water samples were taken and analysed at the beginning and the end of the experiment as well as one time during the experiments, before and after the water exchange (details in paragraph1) | | |  |
| p. Method | | HPLC-MS (QTOF-MS) (LoQ = 300 ng/L) | | |  |
| q. Duration | | 08.08.17 – 05.09.18; 29 days | | |  |
| r. Observations | | Mortality, swimming behaviour, at the end of the experiment body length and body weight were determined and samples were taken for biochemical and histological analyses | | |  |
| s. Results | | Summary table in article | | |  |
| t. Biomass loading | | Mean: 1.9 g/L | | |  |
| **6. Statistical Design and Biological Response** | | | |  |  |
| a. Replicates | Three replicate aquaria per test concentration | | |  |  |
| b. Number of organisms | 10 fish per replicate | | |  |  |
| c. Design | Three blocks, one replicate per treatment present in each block, arranged in randomized order | | |  |  |
| d. Statistical methods | Mortality: nested COX-regression, body length/mass, stress proteins, swimming behaviour: nested ANOVA, histology: likelihood ratio test | | |  |  |
| e. Biological response | None | | |  |  |
| f. Dose-response | None | | |  |  |
| g. Statistical significances | None | | |  |  |
| h. Significance level | α = 0.05, in cases of multiple comparisons adjusted via sequential Bonferroni | | |  |  |
| i. Variability | Not estimated | | |  |  |
| j. Raw data | Provided on request | | |  |  |
